# A digital divide in the COVID-19 pandemic: information exchange among older Medicare beneficiaries and stakeholders during the COVID-19 pandemic

**DOI:** 10.1186/s12877-022-03674-4

**Published:** 2023-01-12

**Authors:** Aaron Castillo, Maricruz Rivera-Hernandez, Kyle A. Moody

**Affiliations:** 1 Brown University, Providence, Rhode Island USA; 2 Department of Health Services, Policy and Practice, Brown University School of Public Health, 121 S. Main St. 6th floor, Box G-121-6, Providence, Rhode Island 02912 USA; 3 Center for Gerontology and Healthcare Research, Brown University School of Public Health, Providence, Rhode Island USA; 4 Communications Media at Fitchburg State University, Room Number: CNIC 316, Fitchburg, MA 01420 USA

**Keywords:** Medicare fee-for-service, Medicare Part C, Advertising, Social media, Fraud, Cognitive dysfunction, Literacy, Television, Internet access, Digital divide

## Abstract

**Background:**

The COVID-19 pandemic resulted in unprecedented challenges for older adults. Medicare enrollment was already an overwhelming process for a high fraction of older adults pre-pandemic. Therefore, the purpose of this qualitative study was to gain understanding from community organizations and stakeholders about their pre-pandemic and during-pandemic experiences while adapting to continue offering insurance advice to seniors, what resources are available to seniors, and what needs to be done to help seniors make higher quality insurance choices in the Medicare program. In addition, we wanted to explore how the COVID-19 pandemic may have changed the ways that these stakeholders interacted with Medicare beneficiaries.

**Methods:**

We employed a qualitative strategy to gain a deep understanding of the challenges that these organizations may have faced while offering advice/counseling to older adults. We accomplished this by interviewing a group of 30 stakeholders from different states.

**Results:**

Every stakeholder mentioned that some older adults have difficulty making Medicare decisions, and 16 stakeholders mentioned that their system is complex and/or overwhelming for older adults. Twenty-three stakeholders mentioned that Medicare beneficiaries are often confused about Medicare, and this is more noticeable among new enrollees. With the onset of the pandemic, 22 of these organizations mentioned that they had to move to a virtual model in order to assist beneficiaries, especially at the beginning of the pandemic. However, older adults seeking advice/meetings have a strong preference for in-person meetings even during the pandemic. Given that the majority of the beneficiaries that these stakeholders serve may not have access to technology, it was difficult for some of them to smoothly transition to a virtual environment. With Medicare counseling moving to virtual or telephone methods, stakeholders discussed that many beneficiaries had difficulty utilizing these options in a variety of ways.

**Conclusions:**

Findings from our interviews with stakeholders provided information regarding experiences providing Medicare counseling pre- and during-COVID-19 pandemic. Some of the barriers faced by older adults included a complex and overwhelming system, a strong preference for in-person meetings among beneficiaries, challenges with technology, and an increased risk of information overload and misinformation. While bias may exist within the study and sample, given that technology-savvy beneficiaries may not seek help from organizations our study participants work in, they show how the current Medicare system may impact vulnerable older adults who may need support with access to high-speed internet and digital literacy.

**Supplementary Information:**

The online version contains supplementary material available at 10.1186/s12877-022-03674-4.

## Background

Every day, about 10,000 older adults who are approaching 65, people with disabilities, and individuals experiencing End Stage Renal Disease (ESRD) have to make choices regarding Medicare coverage [1, 2]. Enrolling in Medicare can be a complex task for older adults and people with disabilities and/or cognitive impairment [3–5]. Older adults who qualified (themselves or spouses contributed to Medicare by paying taxes while working for about 10 years) can sign up for both Part A (hospital insurance) and Part B (Medical insurance) during the initial enrollment period without paying a penalty, starting three months before they turn 65 [2]. Older adults who did not sign up during their initial enrollment period may sign up during general enrollment period (January 1-March 31 each year) and may have to pay a late penalty if they do not qualify for special enrollment (people working past 65) [2]. People with disabilities are eligible for Medicare after collecting Social Security Disability benefits for 24 months and people with ESRD are eligible after receiving regular dialysis treatment or after receiving a kidney transplant [6]. In addition, other Medicare beneficiaries have the option to make insurance changes during open enrollment.

Currently, there are about 63 million people enrolled in Medicare, with 42% of those enrolled in a Medicare Advantage plan [7]. Medicare Part A covers inpatient care in hospitals, and Part B covers outpatient care, doctor’s services, and other medical care. There is also prescription drug coverage (Part D) and supplemental coverage options (Medigap plans). Medicare Advantage – or Part C – often includes Part A, Part B, and usually Part D. In 2021, more than 3,500 Medicare Advantage plans were offered across different markets in the US [8], with the average consumer having about 33 plans to choose from [9]. Similarly, there were about 30 Part D plans available [9]. In addition, coverage, costs, and quality could vary widely among Medicare Advantage plans and Part D prescription drug plans. After the initial enrollment period, beneficiaries can revisit their choices, switch and/or drop plans during Medicare’s open enrollment period (every year during October – December) [10].

Selecting a Medicare plan is not a simple task for any beneficiary [3]. However, older adults have specifically described this as a complex and overwhelming process, as many older adults have no prior knowledge of the Medicare system until they become eligible [11–14]. Confusion about the Medicare program has been reported among older adults from all education and socioeconomic levels, but it can be more prevalent among racial and ethnic minority groups [3]. Older adults have to navigate and process program-specific and insurance-related information mailed/emailed by the Centers for Medicare and Medicaid (CMS), insurance agents, brokers and/or other community organizations. CMS mails older adults the “Medicare & You” handbook, which summarizes Medicare health benefits and options [15]. Medicare-eligible individuals are also bombarded by marketing materials and brochures from insurance companies. In addition, there are large volumes of information available online. For instance, Medicare.gov (*Medicare Plan Finder)* was designed to help older adults make informed choices by reporting locally available options regarding plans’ benefits, out-of-pocket cost, and quality ratings; however, the majority of older adults do not use this website [16]. A great amount of evidence from Medicare-related literature has shown that older adults are overwhelmed with the number and complexity of options offered, especially among those with low socioeconomic status and racial-ethnic minorities [11, 12, 17–21]. Moreover, older adults with cognitive impairment, a high-priority population for the federal government, may be less likely to make optimal enrollment choices [5].

In addition, Medicare beneficiaries may be faced with misinformation impacting their decision-making processes and plan selection [22]. The Information overload problem is exacerbated by the use of social media, platforms that were essential for communicating during the Coronavirus 2019 (COVID-19) pandemic. Given the complexity of the Medicare system and the potential for “information overload” and misinformation, prior literature has found that older adults generally prefer to receive in-person assistance when enrolling or switching plans [16, 23, 24]. In prior years, older adults or family members had different alternatives to seek/receive face-to-face information, including the State Health Insurance Assistance Programs (SHIPs), insurance agents (e.g., UnitedHealthcare, Humana, BlueCross BlueShield affiliates), and insurance brokers. Insurance brokers/agents receive compensation to sell Medicare plans to beneficiaries, whereas the SHIP is a federally funded program that provides Medicare counseling to Medicare-eligible people to make informed choices [25, 26]. However, these programs and organizations, like many other places serving consumers, more likely faced changes as a result of the COVID-19 pandemic [27, 28]. Social distancing guidelines and stay-at-home orders may have presented some challenges for those who benefit from one-on-one in-person interactions, especially among people with physical and cognitive limitations [29–31]. CMS promoted virtual visits using different platforms (e.g., Zoom, Skype, Facetime) to reach beneficiaries [32]. However, a large fraction of older adults may have been unable to adopt some of these technologies due to inexperience with technology or barriers to learning new technology [33, 34]. In addition to individual-level factors, other challenges may have included access to high-speed internet [35, 36].

Medicare enrollment was already an overwhelming process for a high fraction of older adults pre-pandemic [3]. In addition, the COVID-19 pandemic resulted in unprecedented challenges, impacting health and healthcare choices of many older adults [37]. Therefore, the purpose of this qualitative study was to gain understanding from community organizations and stakeholders about their pre-pandemic and during-pandemic experiences while adapting to continue offering insurance advice to older adults (65 and older), what resources are and were made available for older adults, and what needs to be done to help older adults make higher quality insurance choices in the Medicare program. In addition, we wanted to explore how the COVID-19 pandemic may have changed the ways that these stakeholders interacted with older Medicare beneficiaries. Therefore, we employed a qualitative strategy to gain a deep understanding of the challenges that these organizations may have faced while offering Medicare advice/counseling to older adults. We accomplished this by interviewing a group of stakeholders from different states.

## Methods

We used purposive/snowball sampling and reached out to participants from May to June of 2021. Using telephone and/or email, two members of the study team contacted a total of 131 potential participants. Potential participants included organizations (state insurance programs, senior centers, Administration of Community Living, American Association of Retired Persons), insurance brokers, insurance agents, advocacy groups that help provide information regarding Medicare enrollment/disenrollment (choices, Medicare Advantage plans, benefits, out-of-pocket costs, etc.) to Medicare beneficiaries. We excluded any organization, broker, agent that did not serve/help Medicare beneficiaries; organizations or people that did not appear when conducting online searches or via referrals; and anyone who were not able to participate in online interviewing. Out of the 131 potential participants, 20 were closed and/or were understaffed due to the COVID-19 pandemic and did not want to participate in the interview, two mentioned that they were not offering supplemental Medicare or Medicare Advantage options and could not provide input to our study, and the rest did not answer/return our calls, emails, or voice messages.

We interviewed 30 out of the 131 potential participants contacted. Of those 30, 29 were conducted by the first author, with the remaining interview conducted by another team member (see the acknowledgement section). We conducted 27 interviews in the months of July and August 2021 via Zoom. Each Zoom interview lasted 30 min to an hour, and three participants sent their responses in writing, all using structured interview guides (See Additional file 1). The interview questions were based on prior research [3, 16]. We were able to interview people from different regions across the US states. These interviews and responses focused on Medicare enrollment pre- and during-pandemic and disruptions caused by the pandemic. This was the first step to help identify perspectives from stakeholders regarding Medicare enrollment during the pandemic. We recorded each interview and transcribed the recordings.

### Analysis

Transcribed interviews were entered into NVivo. We used content analysis to analyze the interviews. Two members of the study team coded four interviews separately, using priori and emergent codes. We developed priori codes from prior literature regarding Medicare plan choice [3, 16]. Additional codes were developed based on the questions/interview guide. We refined the coding scheme as we analyzed these transcripts and compared/discussed them until an agreement was reached. The first author of the manuscript coded the remaining 26 interviews. These authors met frequently to discuss the analysis and results. Data analyses were conducted using a summary of codes and themes. The authors reached data saturation as no additional themes emerged [38]. For this article, we were interested in presenting data regarding observations about the Medicare program pre- and during-pandemic from stakeholders. This study involved the collection of de-identified stakeholder data. Stakeholders who participated in these interviews did not provide personal information, thus, this study was not considered human subjects research or subject to Institutional Review Board approval.

## Results

Twenty-six interviews were conducted with organizations that provided services for older adults; three participants were insurance brokers, and one interview was conducted with an advocacy organization. The majority of the participants were located in urban areas, and six participants were from rural areas. Eleven participants were located in the Southwest, nine participants were located in the Northeast, six participants were located in the West, and four participants were located in the Midwest.

Every stakeholder mentioned that some older adults have difficulty making Medicare decisions, and 16 stakeholders mentioned that their system is complex and/or overwhelming for older adults. Twenty-three stakeholders mentioned that Medicare beneficiaries are often confused about Medicare, and this is more noticeable among new enrollees. “Choosing coverage can be stressful and nerve-racking, and Medicare gets more complicated every year!” Thus, pre-pandemic, these community organizations and insurance brokers often met with people in-person to go over questions and details. With the onset of the pandemic, 22 of these organizations and insurance assistance programs mentioned that they had to move to a virtual model in order to assist beneficiaries, especially at the beginning of the pandemic. However, older adults seeking advice/meetings have a strong preference for in-person meetings most of the time. Given that the majority of the beneficiaries that these stakeholders serve may not have access to technology, it was difficult for some of them to smoothly transition to a virtual environment. With Medicare counseling moving to virtual or telephone methods, stakeholders discussed that many beneficiaries had difficulty utilizing these options in a variety of ways.

### Theme 1: Choosing a Medicare plan was already a complex task for Medicare beneficiaries pre-COVID-19

Finding the optimal Medicare insurance plan can be confusing. When prompted, one participant said “it's confusing. It's problematic because we're throwing so many choices at individuals from the very beginning. It's not easily understood.” Another stakeholder highlighted that sometimes beneficiaries do not know the difference between traditional Medicare and Medicare Advantage. For instance, one participant mentioned,

“It can take two to three years for seniors to understand what Medicare advantage is. So, they spend the first year to three years kind of floundering around the system, trying to understand how Medicare works and, in the process, they could be missing out on care that they need, they could be paying more, they could be experiencing a lot of frustrations with that interaction between their care and Medicare.”

Another participant from a different organization described something similar:

“I would say all seniors struggle, especially in the beginning when they are getting onto Medicare. They struggle because there are so many choices and so many details within those choices. Even supplements, do you just go with the most coverage, or do you go with something that's a more cost-effective with a little less coverage, but still great coverage? Does it work for you?”

When asked whether participants understood these differences, one stakeholder mentioned that:

“When folks are comparing Medicare Advantage, there are a lot of different supplemental benefits that go with those plans that we do get that information every year,” she went on to say that “we can't just put on a chart and say, ‘Well, here's a comparison of what you'll get with this and this and this.’ Because you really have to delve into the plan documents to get the details, ‘Oh, this has dental. This also has dental." But what are those dental benefits, right? What are those supplemental benefits?’”

One stakeholder mentioned that they have worked with people from all income brackets. She said:

“We've had judges, attorneys, etc. be totally confused about Medicare. We've had people that are working class, lower income, totally confused. So, we need to look at each individual's needs to help them understand what it is they're looking at, what the foundation is, and going from there as to what to do.”

However, participants mentioned that Medicare may be more complex among people with cognitive disabilities:

“It's like you might have a conversation with someone and say like, ‘Okay, here's what we're going to do. Let's do this.’ and then that person may not remember the conversation the next day and so that can be very, very difficult for everyone involved but it's not something that there's always supports in place for and that's something I used to find in my role a lot, is that sometimes people don't have those formal supports in place because they may not want them or for a variety of reasons. So, there are a lot of barriers to making those decisions for folks who may have some cognitive issues or disabilities.”

### Theme 2: Before the pandemic, older adults had a strong preference for in-person meetings, which changed during the pandemic as services were more likely to be offered virtually

Ten of these stakeholders described that most beneficiaries and/or family members have a strong preference for meeting with them in-person despite COVID-19 restrictions. One stakeholder said that:

“The biggest [barrier] would be that we were not able to make in-person appointments, obviously, so this was a major barrier, especially for older adults who do not have access to technology, do not have a computer, things like that so it's possible to do these enrollments and these conversations over the phone, but it's not easy, and it's not something that's preferable to most beneficiaries, and so that was probably the biggest and most obvious barrier.”

Similarly, another stakeholder described that before the pandemic it was not necessary to make appointments. Older adults were able to go to the offices and asked for help. She stated that:

“Oftentimes, our seniors prefer to talk to someone face to face. Our office specifically, and most of our satellite offices, they're [a] walk-in business. People don't necessarily make an appointment; they just show up, kind of like for the Social Security office or something. And so, for some of those people, that was a challenge because they like to know who they're talking to; they like to see that person. So, that's been one challenge for them.”

Some stakeholders described the challenges of helping people who may have had a hearing or cognitive difficulty/impairment, which was even more complex earlier in the pandemic. “It was more challenging for people with disabilities, especially cognitive disabilities. That was more challenging because we couldn't see them face-to-face.” Another stakeholder said:

“Medicare is hard in itself, and having to go through an interpreter and/or one of these services makes our job a little bit more difficult as well. We get through it, but it is very taxing.” In addition, other stakeholders mentioned that for some beneficiaries with cognitive disabilities, this may have been hard because “especially during COVID… if they didn't know how to click a link to get on Zoom where we can screen share, it was all through the phone and all verbal and sometimes words can mesh together.”

### Theme 3: Zoom and/or virtual consultations work for some but not for everyone due to issues of internet literacy, technological adaptability, and access to stable internet or broadband

Two stakeholders discussed that some older adults were comfortable doing virtual meetings (WebEx teams, Zoom) to receive assistance but highlighted the challenges that other older adult faced:

“All of our area agencies on aging, which provides the Medicare service at the local and regional level, were required to use a web platform to do outreach and to provide assistance to Medicare beneficiaries. And so, they use things like WebEx teams, Zoom to provide this assistance… we found that that worked very well again for people that have internet access” then she went on to explain that there were challenges during open enrollment and being unable to assist people in-person “they expected the same level of help this last year… And we couldn't provide it and they didn't have internet access. So, we had to provide it over the phone, which for some people was not a good replacement. They did get somewhat upset.”

Thus, one of the major challenges reported by stakeholders was the lack of access to technology. For instance, one participant mentioned, “there's wide areas…without any broadband. And several places that you can't get a cell signal. So that's something that they'd work hard on.” Nine stakeholders talked about having some form of Zoom meetings/appointments. However, some of them indicated that using a virtual platform was not easy for everyone. One of them mentioned, “I mean, we offered Zoom calls and over the phone, but some, of course, if you have a disability, like hearing disability, sometimes [that] can be difficult if you're not tech-savvy. So, a lot of barriers were involved with that.” Six stakeholders described that some of the older adults they served had issues with internet literacy or access to the internet. For instance, one of the stakeholders talked about this and stated that “some people have less access to that technology. There’re wide areas of [this rural city] without any broadband. And several places that you can't get a cell signal. So that's something that they'd work hard on.”

One stakeholder mentioned that “because it's complex and it is difficult for some people to hear the information over the phone and be able to put it together into a picture that makes sense. Even when we are doing Zoom or WebEx consultations, it's still not quite the same as being able to sit down and have a conversation.” Another stakeholder also described similar issues. For instance, he said that “just [the other day] I did have a lady that is new to Medicare, and so I tried helping her as much as I could over the phone and I'll go over that in the other questions. Unfortunately, I was limited to what she was able to comprehend over the phone.”

Similarly, another stakeholder also mentioned that:

“It's real hard to explain Medicare without having somebody that can look at paperwork, because if they have a question, I can't explain the paperwork over the telephone. You don't have it in front of you, so we can't even look at the same line together. Because by the time I call you, I don't know what your question's going to be.”

In fact, the stakeholders mentioned that sometimes older adults did not know that they have to complete paperwork. One of the stakeholders noted the following: “and if you don't see them person to person, how do you even show them the paperwork that you get from the federal government? Otherwise, they have to find the paperwork… So, if [they] can't find it, how do I help them find it?”.

In addition to the confusion that some older adults were experiencing when trying to understand the intricacies of Medicare, some stakeholders also talked about how some beneficiaries did not want to provide information over the phone. One of these stakeholders described that “[some people] sounded really confused or they were very guarded as far as giving us information, but they really wanted to review their plans or they were new to Medicare or wanted to make changes.” So, they had to figure it ways to convey the information in an easier way and/or bring people in-person, “when we mention, ‘Okay, we can schedule you for an in-office appointment,’ and they're like, ‘Yes, please. I would really appreciate that.’"

When asked how virtual appointments have worked for everyone, one stakeholder responded, “I wouldn't say it's been a complete success, I think there were some people who really needed that in-person appointment but we did what we could to try and stay connected with people and help them enroll during the pandemic and continue to do so.”

### Theme 4: Older adults are overwhelmed with information overload and have received conflicting advice/misinformation, especially during the pandemic

Overall, stakeholders mentioned that they received a lot of phone calls during the pandemic asking questions related to COVID and insurance plan coverage and benefits. One stakeholder described that “we have kept track of the numbers of callers that have called us specifically with COVID questions. And we have over 700 followers that have called us specifically about COVID.”

According to stakeholders, it was not uncommon to hear from beneficiaries that "someone called me and they told me to enroll in this plan and I don't know what I did, and I don't know what I'm enrolled in now…".

Another stakeholder stated that this was often related to the fact that:

“People got phone calls stating that ‘because of COVID, Medicare has to give you new Medicare cards.’ So, then [beneficiaries] give them the Medicare number[s].” Stakeholders mentioned that these companies often sound like they are representing Medicare. Thus, community organizations and other agencies are working on educating people. “Medicare doesn't call you, but I'm able to still talk to them. And we do have people who get changed that did not have a valid, special enrollment period. So, people are not supposed to be able to change just Willy-nilly throughout the year.”

According to some stakeholders, while socially distancing, beneficiaries were spending time at home watching television or other media and may have been vulnerable to false marketing messages. “This past year and a half, with people being home, with COVID, and seeing a lot of the commercials… that Joe Namath commercial that's out right now, so that has sparked people to maybe make changes to their coverage that maybe did not turn out to be as prudent for them.” Another stakeholder mentioned similarly:

“My staff person said, "I grew up loving Joe Namath, and now I can't stand him." Because he does those commercials. And most of what he's talking about isn't available here. So, we get all these questions of are they going to pay for my meals? No, we don't have plans that do that. They're going to do this? No, we don't have plans to do that. And then it also kind of misleading talking about, oh, you can get an extra $140. No, it's not an extra $140. It's only if you're low income and you're getting your 140 back. It's those little things. There's so much on commercials that they are supposed to be helpful and they're just making it more confusing than it has to be.”

Another participant also agreed that these commercials are misleading beneficiaries:

“Making it sound like every older adult in Medicare is entitled to all those benefits. And what I can tell you in [this state], none of our plans, and I mean, none of the Medicare advantage plans provide all those options he talks about or other people that are trying to market and sell Medicare advantage plans. They don't provide that coverage. So, people are being misled.”

Another participant mentioned the rise of third-party companies through Medicare Direct Contracting and/or other managed care entities, “third party companies have definitely increased because I feel like they know people are at home. So, like those phone calls that people are getting is definitely more than what even two or three years ago people were getting, but so are the television commercials and advertisements, so all of that's increased.”

## Discussion

Several interrelated themes emerged from this qualitative study regarding *pre- and during-pandemic* experiences of stakeholders regarding Medicare enrollment. We found that the majority of our participants described the Medicare system as complex and overwhelming before the COVID pandemic; a strong preference for in-person meetings even during the pandemic; challenges with electronic adaptability, technology, and internet access; increased risk of information overload and/or misinformation. While our participants highlight some of the experiences that navigators and other stakeholders have with beneficiaries that need one-on-one support, these findings complement and support prior literature discussing an overwhelming system for Medicare beneficiaries who may or may not use help from navigators while enrolling or reviewing their Medicare options [11, 12, 17–21].

A consistent message across these stakeholders is that the system is overly complicated for both new enrollees and those already enrolled. Due to these complexities, explaining the different Medicare options and choices over the telephone or through video calls was extremely challenging for some stakeholders. SHIPs or other local organizations often use Medicare.gov (*Medicare Plan Compare*) as a tool to present coverage options to beneficiaries. This online tool has recently been redesigned to make it more user friendly for consumers. Medicare.gov now includes a simplify plan menu (excluding drug copayments), allows consumers to make plan comparisons, and displays the drug lists when beneficiaries enter their Medicare IDs [39]. However, according to experts, this tool lacks functionality for the regular consumer, and many older adults may require assistance while using this site [40]. Similarly, stakeholders discussed that despite changes to the plan finder in 2019, there was still difficulty in using the tool when working with beneficiaries. Thus, navigating the tool without someone physically present may be difficult for some older adults. In fact, an overwhelming number of Medicare beneficiaries do not use Medicare tools available to them (e.g., Medicare.gov, 1–800-Medicare) [16, 41]. This is partially related to technological barriers/access to the internet, awareness of these resources or having someone to access these resources for them [9, 16, 40, 41]. In our study, some participants stated that some older adults were comfortable using online tools and communicating with them via Zoom, but the majority of the stakeholders mentioned that the digital divide and tech readiness continue to affect older adults, which is a concern as digital technology becomes more commonplace and requisite for daily tasks.

The pandemic has made the internet an intrinsic part of life for many people [42]. As technology continues to change and become more accessible, older adults may find these online support tools more effective. About 84% of beneficiaries reportedly have access to the internet during the pandemic [43]. However, there are striking differences in socioeconomic status among Medicare beneficiaries. While stakeholders mentioned helping people from all socioeconomic statuses and education levels, technological barriers may have further impacted vulnerable adults. When looking at beneficiaries with incomes of less than $25,000, about one-third of beneficiaries have recently reported not having access to the internet [43]. In terms of technology used, 71% of these beneficiaries appear to rely on smartphones and 66% on computers [43]. As mentioned by our participants, some older adults struggle to use these devices, especially those with cognitive impairment and/or other physical limitations. Of note, for these beneficiaries navigating Medicare.gov on a smartphone could be very unintuitive due to a large number of options available and the longer times needed to shop for plans. Thus, beneficiaries with severe illnesses may have been impacted the most during the onset of the pandemic [44]. In addition, these gaps could have affected the health and insurance literacy, as well as an understanding of basic Medicare concepts, which could have adverse consequences for Medicare beneficiaries as it relates to short- and long-term personal and financial health. Fortunately, when social distancing guidelines were relaxed, stakeholders were able to facilitate in-person meetings to provide that one-on-one needed for those beneficiaries facing personal or technological barriers.

Discussions about the large amounts of information that beneficiaries have to navigate led to conversations regarding susceptibility to misinformation and privacy concerns among older adults. Since the COVID-19 pandemic also increased the risk of cyber-attacks, online fraud, and anxiety among online users [45], these issues further complicated information exchange among stakeholders and beneficiaries. Unfortunately, this may be related to the increasing amount of misinformation and information consumption behaviors among older adults. While a vast majority of Medicare beneficiaries appear to rely on traditional news and government sites for health-related information, close to one-third of beneficiaries obtain information from the internet or social media [43]. Along with this, stakeholders mentioned that beneficiaries had been victims of deceptive and manipulative marketing materials, commercials on television, and people selling plans that overpromised coverage, out-of-pocket costs, and benefits. This issue was described by the majority of the stakeholders that we interviewed. Prior administrations have focused on reducing premiums and improving benefits [46]. Thus, news and trade sites have reported that Medicare Advantage advertisement appears to be increasing in recent years as insurance companies are working on improving their enrollment numbers [47, 48]. Many stakeholders shared this view. While televised ads for health insurance has the potential to encourage engagement among consumers and plan enrollment [49], they could mislead vulnerable consumers who have less understanding of insurance literacy to believe that these plans are “free” (zero premium, zero deductible, and zero co-pays) [50]. In addition, stakeholders mentioned that these commercials are generic, while Medicare Advantage plans and benefits vary by county [8]. While plans in some regions may have the benefits advertised, some beneficiaries ended up enrolling or switching to these plans without understanding the fine print and lost some essential services that were previously covered.

Our study has some limitations. First, this was a convenience sample of people who provide Medicare coverage counseling to Medicare beneficiaries. Participants included people that we identified via online searches and referrals. Our results may not be generalizable to a broader population of insurance brokers/representatives or advocacy groups. Second, due to our sample size, we were unable to compare rural–urban differences within the same state. Stakeholders in urban areas may have different experiences than those in rural areas. Third, we included stakeholders who were more likely to participate and were able to participate in online interviewing. Finally, the stakeholders that we interviewed are more likely to work with older adults that may prefer receiving one-on-one support from an insurance broker/agent, an organization or a person not affiliated with Medicare. Thus, these stakeholders may be more likely to interact with older adults who may be less technologically-savvy and comfortable using and interpreting Medicare.gov and/or other decision support tools, getting help online or over the phone, and have limited or poor access to computers or mobile devices and high-speed internet. Additional research should use a more diverse sample of stakeholders, explore differences between Medicare beneficiaries and stakeholders from different markets, and with different levels of socioeconomic status, health literacy and access to technology, and whether the COVID-19 pandemic impacted the choices made by Medicare beneficiaries pre-pandemic and today as it begins to wane.

Our results have policy implications. While the availability of decision support tools and technology-savvy consumers may increase over time, the Medicare system is very complex for many beneficiaries as specified by these stakeholders. In addition, many Medicare beneficiaries and older adults currently face technological barriers, information overload (including exposure to changing forms of misinformation), and choice inconsistency [20, 34]. Thus, Medicare literacy, as well as providing one-on-one counseling and assistance to Medicare beneficiaries, should continue to be an important task for the federal and local governments until they system is less overwhelming for beneficiaries. Unfortunately, unbiased organizations like SHIP, rely on volunteers and funding from the federal and local governments. It is not uncommon to see letters from the National Council on Aging advocating for funding for SHIP, including the one sent in February 2021, in the middle of a pandemic [51]. Thus, limited funding for these organizations may have negative consequences for the millions of beneficiaries faced with complex choices.

## Conclusions

Findings from our interviews with stakeholders provided information regarding experiences providing Medicare counseling pre- and during-COVID-19 pandemic. Some of the barriers faced by older adults included a complex and overwhelming system, a strong preference for in-person meetings among beneficiaries, challenges with technology, and an increased risk of information overload and misinformation. While bias may exist within the study and sample, given that technology-savvy beneficiaries may not seek help from organizations our study participants work in, they show how the current Medicare system may impact vulnerable older adults who may need support with access to high-speed internet and digital literacy. With the higher number of older adults approaching 65 and Medicare beneficiaries, complexities of the Medicare system, and technological barriers, one-on-one assistance will continue to be an essential service that should be available to anyone who needs this service.

## Supplementary Information

**Additional file 1:** Interview questions.

## Data Availability

The datasets used and/or analyzed during the current study are available from the corresponding author upon reasonable request.
