# Supplementary material for: A digital divide in the COVID-19 pandemic: information exchange among older Medicare beneficiaries and stakeholders during the COVID-19 pandemic
Source: BMC Geriatr. 2023 Jan 12;23:23. doi: 10.1186/s12877-022-03674-4 (PMC9836741; doi:10.1186/s12877-022-03674-4)
Supplement: Supplementary file 1 — Additional file 1: Interview questions. [file 12877_2022_3674_MOESM1_ESM.docx]

**Additional file 1, Interview questions**

**Introduction: Explain study procedures and details to participants, including audio recording.**

This interview will take about 30 – 60 minutes, depending on how much you would like to share.

I would be happy to answer any questions that you may have for me before we begin.

This study is about how you may help people enroll for the first time, switch and disenroll from their Medicare Advantage (MA) program.

There is a large number of choices in the Medicare program and choosing a plan can be quite difficult for Medicare beneficiaries. We have found that community organizations and other stakeholders (e.g., government agencies, leaders in churches, senior centers) are great resources for people to find more information about MA plans. We are trying to identify barriers in decision-making and plan choice and to generate input on resources needed to create the decision-support tool that would help to simplify and make this process easier for older adults (65+).

***Warmup: To begin I would like to know a little about you.***

- How are you doing today?
- What do you like to do in your free time?

***Helping with enrollment/switching***

Now let’s go to the interview. I would like to start by talking about Medicare enrollment and switching.

- What is your opinion about Medicare enrollment? Are there any anecdotes that you may want to share?
- When do older adults/beneficiaries have more trouble/problems choosing a plan? Could you provide some examples?
- In your opinion or experience, who needs more help when choosing a plan? Are there particular features that older adults are more interested in learning about (e.g., costs, benefits, out-of-pocket costs, providers, network, etc.)?
- What kind of advice do older adults/beneficiaries need to make a choice? Is this for enrollment/disenrollment?
- Could you describe the steps that people use to find information about MA plans online?
- Are there specific resources that would be more useful to older adults/beneficiaries?
- What online resource would be best to provide advice regarding MA?
- What are the basic misconceptions that older adults have when making a choice?
- What is the most difficult part of this process?
- Have you seen older adults use CMS tools?
- Are some resources more useful than others?
- Have you helped older adults that may have cognitive disabilities and/or other limitations? What are some additional barriers that these beneficiaries face? How can CMS make choices easier for them? What are some resources that would be helpful for these beneficiaries?
- Do older adults talk about things that they do not like about their plans?
- Are there any negative experiences?

***COVID-19***

- Could you tell me, what are some of the barriers that older adults have faced during the pandemic when shopping for plans?
- Have you implemented anything specific to address this?
- Have you switched to an all virtual model?
- If so, how have you adapted services to fit this model while still making them accessible to older adults?
- Were you offering any services during open enrollment?
- Were there any questions that older adults have during the pandemic?

***Help/support tool***

- Please tell me what helps older adults to make their insurance choices.
- Do you older adults choose the best option available to them?
- Do older adults find insurance terms confusing? Which ones?
- Do you (or others) help them understand what the terms meant?
- What are the things that older adults care about (e.g., quality, ratings, etc.?)
- How would you design online educational products facilitating information about MA plans?
- Would something that target decision making would be useful among older adults (perhaps an app)?
- What changes would you make to Medicare Plan Finder? Or how would you use this tool to help older adults?
- How can we make this website more accessible/usable to people?
- Are there other platforms with a greater application for MA choice awareness?

***Exit questions:***

- What advice would you give others enrolling for the first time? What information is most important to deciding which plan to choose and what resource should older adults use?
- That was my last question. Is there anything else I should have asked or that you would also like to say?

Note: The interview questions were based on prior research.

Rivera-Hernandez M, Blackwood KL, Moody KA, Trivedi AN. Plan Switching and Stickiness in Medicare Advantage: A Qualitative Interview With Medicare Advantage Beneficiaries. Med Care Res Rev. 2021 Dec;78(6):693-702. doi: 10.1177/1077558720944284.

Rivera-Hernandez M, Blackwood KL, Mercedes M, Moody KA. Seniors don't use Medicare.Gov: how do eligible beneficiaries obtain information about Medicare Advantage Plans in the United States? BMC Health Serv Res. 2021 Feb 15;21(1):146. doi: 10.1186/s12913-021-06135-7.
